# Mixed blessings: A qualitative exploration of mothers’ experience of child care and feeding in the rapidly urbanizing city of Addis Ababa, Ethiopia

**DOI:** 10.1371/journal.pone.0207685

**Published:** 2018-11-20

**Authors:** Hanna Y. Berhane, Eva-Charlotte Ekström, Magnus Jirström, Yemane Berhane, Christopher Turner, Beatrix W. Alsanius, Jill Trenholm

**Affiliations:** 1 Department of Women’s and Children Health, International Maternal and Child Health, Uppsala University, Uppsala, Sweden; 2 Addis Continental Institute of Public Health, Addis Ababa, Ethiopia; 3 Department of Human Geography, Lund University, Lund, Sweden; 4 London School of Hygiene and Tropical Medicine, London, United Kingdom; 5 Department of Biosystems and Technology, Swedish University of Agricultural Sciences, Alnarp, Sweden; Christiana Care/University of Delaware, UNITED STATES

## Abstract

Many studies have drawn attention to the vital role mothers have in safeguarding the health and nutritional wellbeing of their children. However, little is known about mothers’ experiences and the challenges they face in fulfilling this role in rapidly urbanizing cities in Africa. This study aims to explore child care and feeding practices of mothers with children under five years of age in Addis Ababa, Ethiopia. This qualitative study was conducted using a semi-structured interview guide. A total of thirty-six interviews were conducted with purposively selected participants. All interviews were audio recorded, transcribed verbatim and translated for analysis. We used a thematic analysis approach, which was guided by a resilience framework. The findings are presented as three major themes. 1) ‘Mixed blessings-balancing motherhood’s expectations’. While mothers identified positively with the social recognition and sense of fulfillment of being a ‘good mother’, they were ambivalent/torn about earning the necessary income from outside work and fulfilling their duties at home. 2) ‘Instabilities due to rampant urban sprawl’. While women expressed a keen desire to balance work and motherhood, the disintegrating social capital, due to large in-migration, market fluctuations and abrupt/forced resettlements to new housing units had left mothers without support for childcare, stressed and exhausted. 3) ‘Anchored by faith: a source of resilience to cope with adversities’. In the face of the multiple adversities, mothers cited their strong faith as their most reliable foundation for their resilience. In summary, the societal and environmental changes accompanying the rapid urbanization in low income settings makes combining child care and working outside the home very challenging for mothers. As a result they suffer from fatigue and feelings of isolation. Efforts to improve child feeding and care in urban low-income settings need to consider context appropriate strategies that support mothers with small children.

## Introduction

Childhood malnutrition is a preventable condition that still contributes to more than half of all deaths in children under five years of age globally; the majority of which occurs in low-income settings[1]. In Sub-Saharan Africa chronic under nutrition affects 1 out of 3 children under five [1,2]. Similarly, in Ethiopia 38% of children under five are stunted [3,4].

According to UNICEF, adequate and appropriate child care and feeding practices are considered key determinants of child nutrition, growth and wellbeing [5]. The concept of “Child Care” encompasses: provision of time, attention, support, and having skills to meet the physical, mental, social, and nutritional needs that are critical for the proper growth of children [5–7]. Child care in most societies is ‘women’s work’. They become the primary caretakers of children and bear greater responsibility of ensuring their children’s health and survival[8]. Child health outcomes are positively correlated with improved status for women [9].

Motherhood is highly desired, prized and considered integral to a woman’s self-worth in Africa[10]. However being a mother is also challenging and accompanied with coping with the inherent physical and emotional changes, child care responsibilities, working both at home and outside to fulfill the needs for their family, as well as meeting the social expectations of the mothering role by which women are judged as good or bad mothers [11–15]. A mother’s wellbeing depends on how she conceptualizes her role, for example, always being available for the children and ignoring her own needs to balancing her needs with those of the children[16]. Studies have shown that maternal wellbeing and self-efficacy can also influence infant feeding practices [17,18].

Constructs like “self-efficacy” “women’s status”, “women’s empowerment” and “women’s autonomy” have been used in different studies together and/or interchangeably to explain its association with child outcomes [8,19–21]. This inconsistency has made it difficult to come to any empirical consensus as to what one is measuring and its impact. Though the role of mothers in determining the nutritional status of their children is undisputable, its specific influence requires even further exploration to determine its role in different socio-cultural settings [20].

Urban dwelling is one such socio-cultural context. Rural to urban migration has been considered advantageous primarily due to better economic opportunities, and also due to ease of physical access to healthcare facilities. Many sub-Saharan African countries are undergoing this rapid urbanization which in turn is straining their infrastructures and fueling poverty[4,22–25]. In low-income settings, women take advantage of their social capital in raising children[26]. Social capital encompasses social relationships, networks, and values that facilitate collective action for mutual benefit[27]. In rapidly growing urban areas with its frequent movements and resettlements, the social capital risks destabilization [26] and mothers lose their established support systems. While research globally focuses on the links between women’s status and child nutritional outcomes, less emphasis is placed on the lived experiences of mothers living in rapidly urbanizing settings. Previous qualitative studies were either mostly from South Asia or often focused on rural populations. Considering mothers are vital to sustaining the health and growth of future generations, it is imperative to understand how mothers are managing to fulfill their important roles of child care and feeding, in these fast growing African cities[23]. Such studies are rare in Africa thus this study addresses an important gap in exploring mothers’ lived experiences of child care and feeding in a rapidly urbanizing Addis Ababa, Ethiopia.

### Theoretical perspective

Cities often have diverse, dynamic, and complex social contexts that are difficult to understand fully. In the context of this study in Addis Ababa, women were faced with many challenges in their quest to care for their children. We chose Kumpfer’s resilience framework (2002) [28] as a theoretical lens in which to view women’s personal experiences because it had the ability to capture a holistic perspective of the mother’s daily struggle when caring for her children. According to that framework, resilience is a dynamic transactional process resulting in a relatively optimal adaptation to a way of life which is initially triggered by a stimulus (a stressor) and then passes through different buffers such as one’s psychological, emotional and physical capacities as well as environmental contexts[28]. There are many definitions of resilience but for the purpose of this paper we concurred with Ungar’s definition: “In the context of exposure to significant adversity, resilience is both the capacity of individuals to navigate through psychological, social, cultural, and physical resources that sustain their well-being, and their capacity individually and collectively to negotiate for these resources in culturally meaningful ways”[29]. In this particular paper, we focus on the individual mothers’ self-assessment. In an upcoming paper we explore in depth, how she operationalizes her actions within the food environment[30].

## Methods

### Study population and settings

This qualitative study is based on phenomenological foundations, which hold that the lived experiences of a person can be understood through their utterances. Interviews were conducted with urban mothers’ in the Lideta sub-city of Addis Ababa during March—November, 2016. Addis Ababa, is the capital of Ethiopia and one of the fastest growing cities in the continent. Due to the promise of better job opportunities, the city has become a melting pot for people coming from all parts of the country seeking work. The current population is estimated to be more than four million [31].

Addis Ababa has a fertility level below replacement level [3], yet continues to grow due to in-migration from rural areas. The construction/building sector provides one of the largest employment opportunities for unskilled labor [32]. When the city was founded in 1886 there was no plan, subsequently, it is currently under heavy reconstruction and development in order to modernize. In this process, much of the old parts of the city have been demolished and inhabitants are being relocated to completely new residential areas, causing severe disruptions in their sense of community, neighborhood values and social capital [33,34].

Addis Ababa is divided into ten sub cities. We conducted our study in Lideta sub-city which has an estimated 224,000 inhabitants with a population density of 16,256/km^2^ [31]. Lideta sub-city was selected for our study due to the high population density and the diverse socio-economic composition which served as a proxy microcosm of the larger metropolis. Data were collected from purposively selected mothers from all ten districts that exist in Lideta sub city.

### Data collection procedure

Thirty six in-depth interviews were conducted with mothers who have at least one child in the age group 0–59 months, and who were permanent residents of the city (lived in the city for at least 6 months). The purposive sample included a diverse group of mothers based on: the age group of their child (0–5 months, 6–23 months, 24–59 months) in order to capture the various nutritional needs, numbers of children and mothers with different employment statuses. The researchers identified respondents with the help of health extension workers, who had a list of residents within their catchment area.

Interviews were conducted in Amharic (the national language) using an interview guide. All interviews were audio recorded with the mother’s consent. Experienced research assistants, all with master’s level education, conducted the interviews after attending a two-day training workshop. The first author, an Ethiopian who is a permanent resident of the city, provided the training and stayed on site during the whole data collection period to supervise the process and provide regular feedback. The average time to complete the interviews was 47.8± SD 20.25 minutes. Initially thirty interviews were completed. After a preliminary analysis and discussion with the research team, an additional six interviews were conducted by the first author to follow-up certain issues in more depth.

### Data analysis

Data analysis commenced alongside data collection. The audio recordings were first transcribed verbatim in Amharic and then translated into English for the analysis. The thematic analysis approach as per Braun and Clark [35] was utilized. It began with a thoughtful reading of the transcripts through a theoretical lens of the resilience framework mentioned previously [28, 35]. Two of the authors independently read and re-read the transcripts to understand the data and generate initial codes. Through a series of discussions, these codes were refined, mapped and organized into themes which were further redefined through several iterations until the research team reached consensus on the meaning and interpretation of the thematic areas. The draft findings were also presented to culturally competent academics and stakeholders to confirm more contextual understandings. The analysis was concluded after a thorough discussion among all the authors/researchers.

### Ethical considerations

Ethical approval for this study was obtained from the Institutional Review Board of Addis Continental Institute of Public Health. Before approaching individuals, the necessary permission was sought from Lideta sub-city and all district (Woreda) health offices. All interviews were carried out in the privacy of the mothers’ homes; necessary precautions were taken to avoid intruders or avoid any situation that could make the mother uncomfortable. Verbal informed consent was obtained before starting each interview which included permission to use audio recorders. Data were then kept securely and only accessible to the research team members.

## Results

As shown in Table 1, the mean family size was 5.1 (SD±1.8). The majority of the mothers had more than one children (75%) and the mean age of children in the study was 18.6 (SD±16.1) months. At the time of the interview 39% of the women were engaged in income generating activity. Though most of the mothers had formal education, only five of the mothers had higher level education (diploma and above).

**Table 1:** Participant’s main characteristics.

Child’s mean (SD) age in month	18.6 ± 16.1
Family size mean (SD)	5.1 ± 1.8
Marital Status	
Single	2 (5.6)
Separated	2 (5.6)
Married	32 (88.9)
Parity	
Primiparous	9 (25)
Multiparous	27 (75)
Involved in income generating activities	
Yes	14 (38.9)
No	22 (61.1)
Educational status:	
Can't read or write	5 (13.9)
Primary school	9 (25)
High school	17 (47.2)
Diploma and above	5 (13.9)

The findings of the study are summarized in three main themes: “Mixed blessings: balancing motherhood expectations”, “Instabilities due to rampant urban sprawl” and “Anchored by faith: a source of resilience to cope with adversities”. Direct quotes from the transcripts are used to portray the mothers’ own experiences. Numerical labels have been used to represent the respondents ensuring anonymity and confidentiality.

### Mixed blessings: Balancing motherhood expectations

The women conveyed the difficulties in balancing their dual roles of being full time mother at the same time attempting to contribute to the family’s economy. They often expressed how they positively identified with the role of motherhood despite the all-encompassing demands. They highlighted the social recognition and sense of hope they felt when caring for their children. They described their children as the center of their universe, their joy and hope for a brighter tomorrow:

*I1: “when my children say …if one of us reach to a better position in the future the time will come for you…this will all be history*. *I hold on to that thought… I relax and dust myself up”*

*I2- “… Now I live for my son, I do everything for him*. *Everything I do revolves around him…”*

*I30- “…I will sacrifice myself for my children* … *I will do anything for them as long as I am alive”*

Growing economic challenges have posed a dilemma to the ideal motherhood. The women agreed that taking income earning jobs enables them to support their families financially, save money, and purchase desired material goods for their children; yet, mothers wondered whether working outside the home was worthwhile considering the deteriorating quality of care children would receive in their absence.

*I9*: *“…It is hard to say, a mother working and earning better is good, but it is always a problem getting the right help”*

This mother verbalizes her worries about leaving her child and the potential dangers:

*I10: “No mother wants to leave her children with someone else by choice*. *Unless it is a must… unless they need the income to survive…that is the only reason why they leave their children… to work and attend other social obligations. … Otherwise so many things could happen… the child may fall down and end up disabled. They (alternative caregivers) might feed them food that was not prepared in hygienic way…”*

Participants expressed that the sense of trust that once existed among neighbors or other hired help has been fractured or broken. This mother’s quote captures the precarious nature of having to leave one’s child in the care of another person.

*I31: “…the boy was born big and he was fat (meaning well nourished) when his mother looked after him …then when she decided to go back to her work … a baby-sitter started to take care of him… the parents bought different kinds of things for their child …they provided lots of milk but she [referring to the baby sitter] drinks it for herself… then the baby started to lose weight and even became ill*.

Mothers with family around to help them with child care and those with the ability to get flexible working hours were said to have better capacity to engage in paid work:

*I34: “I got pregnant with my child and stopped working at the beauty salon*. *The father of the baby doesn’t help, so it was my family who supported me with child care …But now I hired help…the work at the beauty salon was from dawn to dusk…so (I) switched to work as a waitress instead”*

Financial support from families also helped mothers cope with economic challenges as expressed here:

*I35: “…since I was not working my mother was supporting me financially*.*”*

On the contrary, the absence of key family members results in loss of social support leaving mothers under enormous pressure. It was not just the mother’s relocations they spoke about but the migration of the people around them. Relocations of their husbands or other close relatives disrupted the family structure eroding essential social capital.

*I2: “…previously when my child was young*, *my sister used to take care of him, that was a great help…she is now abroad”*

Mothers expressed that they are dependent on remittances sent to them by their migrant husbands working abroad or elsewhere. The income was said to enhance food purchasing ability but absence of the father forced the mothers be responsible for “everything else”, restricting her mobility as well as her engagement in social activities.

*I22: “I don’t have a job, but my husband is working in Arab countries… the main source of income for the family (a family of nine) … is what my husband sends to us*.*”*

*I8: “my husband is barely with us; he travels to Djibouti often, he is a heavy truck driver… the main source of income for the family is what my husband sends… I am now actually waiting for my sister to come and babysit my child so I can go to the bank to receive what he sent*.*”*

### Instabilities due to rampant urban sprawl

This theme describes how mothers in rapidly urbanizing cities experience stress related to the disruption of their neighborhood and the social support it provided due to resettlements of the family to other areas as a result of this ongoing reconstruction of the city.

Mothers voiced their concerns about being forcibly relocated from their familiar neighborhood. Resettlement not only dismantled their social network but also affected their livelihood due to increased house rents and additional transport expenses as children move far away from their school and parents from their work. For some mothers the move entailed not having employment opportunities:

*I19: “…I used to have a better income when I was living in ‘American Gibi’ (the place she used to work). I used to earn around 300 Birr working at multiple places plus lunch was provided, my child got better meals there …but that is not happening now. Since the area is to be demolished… people who can afford to hire …have already left the area…so it is difficult to get such kind of work*.*”*

This mother’s story relates to how resettlement due to the city’s renovation leaves her in a compromising situation; it rendered her homeless and unable to get stable housing of her own, forcing her to live in uncertainty:

*I28: “When we had to resettle to a different area … I was not even 40 days post- delivery. I was told the house is about to be demolished and I have to move out. I had nowhere to live… I contemplated about moving to the new areas, but the cost of house rent is high and the money I have will not be enough… My salary is 700 birr*, *in the new areas rent is 800–1000 birr therefore moving was impossible”*

### Anchored by faith: A source of resilience to cope with adversities

In dealing with the pressures imposed upon the participants by social and economic changes, the mothers in this study stressed how their faith was a fundamental part of their daily life; how it helped them make sense of the environment and to accept their situation. Throughout the interviews the mothers voiced that their faith was a source of hope, strength and a shelter for emotional refuge.

*I1: “…sometimes I wonder what I will do if the kids get sick, I don’t have 5 cents… I just pray to God to help me. You need to set aside some money for emergency… but I can’t…I worry but then I say as God’s will… and leave it all to him*.

This mother’s faith sustains her in the face of risking her children’s health because she cannot afford bottled water.

*I30: “…they drink pipe water. I have never bought the bottled water for them, not even when they were infants… people say the kids will get ill from drinking untreated water… but since I can’t afford to buy bottled water, I say God knows and give it to them*.*”*

This mother’s reflections depict the social isolation many women endured in the city and the importance of the church:

*I32: “I don’t share (my problems) to anyone. I just go to church and find a quiet place and talk to God … like a crazy person, just to let it out of my system and then everything disappears. I don’t have friends; I don’t have someone that I consider close to share my problems … I don’t really have anyone*.*”*

## Discussion

This study aimed at exploring the experiences of child care and feeding amongst mothers with children under five years of age in a rapidly growing city in Ethiopia. The findings revealed a number of challenges mothers face in their efforts to provide adequate care and food for their children. Though motherhood was described as a welcomed milestone in a women’s life, provision of adequate child care and food remains a formidable challenge to women. The social and self-imposed expectations about being a “good mother” coupled with a rapidly changing social context due to rapid urbanization further exacerbates their fatigue. Despite that, these mothers exhibited profound resilience and an unwavering faith to be “everything” they could for their children.

The theme “Mixed blessings”, vividly depicts the dilemmas that women were faced with in trying to balance their multiple roles. Being a full-time-stay-at-home mother or an income-earning mother loomed large as an irreconcilable choice. The dilemmas reported by the mothers in this study are consistent with the earlier studies which highlighted the importance of considering the women’s desire to achieve a higher status in society by being productive outside home as well as establishing their sense of fulfilment in life as mothers [13–15]. Although the burden of child care may be better accommodated in rural settings, the urban lifestyle expands the domain of women’s responsibilities beyond child care and household chores [36]; women are expected to join the workforce and contribute to the family’s income[37]. The combination of these stressors compromises women’s wellbeing which in turn has been shown to affect the health and nutritional status of their children [38].

Our findings show that mothers are faced with multiple environmental and social stressors in their efforts to provide adequate food and care for their children. Kumpfer’s resilience framework shines a more nuanced light upon the process involved in adapting to such stressors/challenges[28]. The adaptation process, according to this framework, happens over time as the individual seeks support/resources from their environment (as in social capital) or from within themselves, relying on cognitive, emotional and spiritual capacities. Mothers in this study demonstrated various abilities to adapt depending highly upon their available social capital. Some of the most disruptive events that affect women’s social capital include out migration of family members for economic reasons and resettlement to new neighborhoods.

The rapid city growth and ongoing reconstruction to modernize the city has demanded resettlement of families to different neighborhoods. Moving away from the familiar neighborhood forces people to lose their social networks and in some cases their access to market, affecting their source of revenue. Several studies that focused on urban resettlement in Ethiopia have criticized the reconstruction process for neglecting the social and economic welfare of its residents, especially the poor [33,34]. This was evident in our study.

Many cities in Ethiopia entertain large volume of in- and out-migration of populations [38]. These migrations are often related to economic activities and involve young and productive segments of the population, sometimes including the breadwinner of the family. These population movements have a ripple effect which contributes to the loss of support to the mothers, financially, emotionally and otherwise. Studies from South America found that although families get financial benefits from the remittances sent back to them, the absence of supportive family, especially when the father is absent, reflects negatively on their families’ wellbeing [39,40]. The old African saying “it takes a village to raise a child” which signifies the collective responsibility of child care may still be applicable but is challenging to fulfill in the urban setting in flux. The continually dissolving social support systems are forcing urban mothers to shoulder the full pressure of child care alone. Our study found mothers to be often stressed out and overwhelmed by the scale of these changes. Previous studies have shown that harnessing social support is instrumental in creating opportunities for mothers to work, earn an income [14] and improve their child feeding practices [41].

As mothers fulfill such a key role, alleviating women’s stressors is of great importance for any child health and nutritional improvement programs. The need is most acute in rapidly growing cities in low income settings and calls for innovative approaches to support mothers who are enduring very taxing situations. One such initiative could be strengthening mother-to-mother support networks, which has been shown to be successful breast feeding promotion initiatives [42]. Additionally, supporting the establishment of low-cost center-based child care services could ease the mothers’ burden. For example, by allocating designated land or space, it would be economically beneficial for the country’s economic growth and families’ as it would encourage women’s participation in the labor market. The introduction of such interventions should be done with caution and with further contextual investigation as studies from low and middle income countries did not yet yield conclusive results on the impacts of subsidized daycares on children’s wellbeing [43,44].

In our study context, despite encountering multi-faceted challenges mothers showed a great deal of determination to offer the best they could for their children. The most prevailing element that mothers believed had sustained their resilience was their strong faith. This is consistent with previous studies [45]. One may wonder, however, whether faith alone is sufficient in the face of a shattered collective, happening in urban settings. Pargament et al. [46], speaks of religious affiliation as having multiple meanings to people: providing a strong sense of hope that God will provide their daily needs and creating a social network through group worship. The latter was not evident in the context of our study. Utilizing the existing faith-based organizations, (i.e. worship meeting places) as a hub for social networking, for providing health and wellbeing information as well as psychosocial support would be worth exploring.

The findings of this study have revealed serious implications on the wellbeing of families, particularly women and children. It would therefore be critical to understand the context in which mothers live in, in order to effectively design and implement child survival interventions that would not further increase the mother’s burden. A study in southern Ethiopia demonstrated that just provision of nutritional supplement for malnourished children does not solve the problem as mothers reported sharing and /or selling the nutritional supplement to meet the demands of other family members when there was lack of food[47].

This paper makes important contributions to understanding the context and challenges that women face in caring and feeding their children in a rapidly growing urban setting in Africa. That said, it is both a strength and a limitation in that this study is exclusively focused on the experiences of women. It illuminates the rarely researched urban mother’s ability to manage day to day care but does not provide perspectives from other contributing family members. Thus, future studies would be wise to include fathers’ perspectives to more fully understand the challenges families in rapidly expanding cities face. This is of paramount importance considering the changing role of fathers in overall child care and feeding alluded to in this study.

Another limitation reflected upon, is the fact that not being able to feed and/or care properly for a child may be a sensitive issue for mothers to comfortably speak about. However, women seemed to want to share their experiences. Giving voice to their struggles and feelings of isolation seemed to have been a welcomed opportunity. The participants’ willingness to speak and their noted relief after the interview may have been a result of the female interviewers/first author’s ability to relate to what they were saying and the ease of rapport created during the interview. Research can provide a forum for those voices not often heard.

To ensure trustworthiness in qualitative work, ongoing reflexivity and a critical reading of results represents a form of rigor. Field notes were taken and many discussions with the other authors/researchers were held in order to discuss the themes which have been presented in different forums. All themes were scrutinized before they were finalized. Two of the authors are native Amharic speakers and their contextual knowledge in terms of linguistics, cultural references and colloquial statements contributed greatly to the credibility of the study.

## Conclusions

This study’s findings contributes to a more nuanced understanding of the unique challenges mothers encounter in the rapidly growing city in Africa. Mothers are overburdened by child caring and feeding responsibilities in the context of declining social support systems and evolving gender roles, depending heavily on their faith for support. In rapidly growing cities in Africa, testing and promoting a range of evidence-based mother support systems is acutely needed to compensate for the disappearing traditional social support systems normally relied upon in child caring and feeding.

## Supporting information

S1 File S1_interview guide. (DOCX)
