# Supplementary material for: Mixed blessings: A qualitative exploration of mothers’ experience of child care and feeding in the rapidly urbanizing city of Addis Ababa, Ethiopia
Source: PLoS One. 2018 Nov 20;13(11):e0207685. doi: 10.1371/journal.pone.0207685 (PMC6245682; doi:10.1371/journal.pone.0207685)
Supplement: S1 File — (DOCX) [file pone.0207685.s001.docx]

**Interview Guide:**

Good morning/ Good afternoon; before I start with my question can you please introduce yourself to me?

1. Please tell me a little about yourself and your family:

*Probe points: Family size, number of children, age, marital status, occupation, educational status, length of residency, relatives, and main source of income?*

1. Thank you. Now we are interested to know what your day to day activity looks like. If it helps, think about yesterday or today and tell me how your day goes by starting from the morning (from when you woke up in the morning) to the evening?

*Probe points: does mother engage in any income earning activity, how does she manage her time, does she have the freedom to move around and freely make decision on how to plan and execute her day?*

1. Household food consumption: What type of food do you and your family commonly consume?

*Probe points #1: take yesterday as an example and tell me what time of food where consumed within the household, any reasons why you choose these food items? Which types of food are mostly affected by the reasons mentioned above?* *Are there any food this family rarely/never eat? What are they and for what reason?*

*Probe points #2: does you or any member of your family eat outside the house? On what occasions?* *Do they ever consume any street food? If yes; what are the commonly consumed foods?*

1. Household food source: Who does the food shopping for family?

*Probe points #1: Do you buy all food items in the same place? Any reason why you chose the different outlets to buy different food stuff? In what circumstances do you change where you purchase your food?*

*Probe points #2:* *Do you get food from sources other than purchase? Home gardening? Or other means? What do you do when food runs out?* *Are there any support systems available? How do you cope during such trying time? What does other families in your neighborhood do?*

1. Child care practices: Who is the primary care taker your child?

*Probe points #1:* *Do you get support in child care? It could be neighbors/other family members? Have you ever observed changes in child care when someone else takes care of your children?*

*Probe point #2: who takes care of children when the mother is working mother? What are your personal experiences? What have you observed in your neighborhood? Have you observed any changes in child care practices over the years? Or even from when you grew up?*

1. Child feeding practices: Who buys and prepares food for your child?

*Probe points #1:* *What is the role of father in buying and preparing food for children? Is the food for child prepared separately?*

1. Child feeding practices: What types of food do you usually feed your child?

*Probe points #1:* *Take yesterday as an example and tell me what your child had from the moment he/she woke up until he slept in the evening. tell me what he/she had for breakfast, snacks, and drinks in between?*

*Probe points #2: Any reasons why you gave those food items to your child?*

*Probe point #3: what are the popular foods given to children in your area? What street foods are available? Which are commonly given to children?*

*Probe point #4: Try to remember of your own childhood and tell us what is different from the way you were raised? Has the child feeding and care provision changed from when you were a child? Or from when you raised your older children*

*Probe point #5: Is it common to give treats to children? What are some of the common ones? What are some of the common circumstance that you give treats to your children?*
